# IL-10 and TGF-β Induced Arginase Expression Contributes to Deficient Nitric Oxide Response in Human Visceral Leishmaniasis

**DOI:** 10.3389/fcimb.2020.614165

**Published:** 2021-02-18

**Authors:** Manu Kupani, Smriti Sharma, Rajeev Kumar Pandey, Rajiv Kumar, Shyam Sundar, Sanjana Mehrotra

**Affiliations:** ^1^ Department of Human Genetics, Guru Nanak Dev University, Amritsar, India; ^2^ Department of Medicine, Institute of Medical Sciences, Banaras Hindu University, Varanasi, India; ^3^ Research and Development Division, Thermo Fisher Scientific, Bangalore, India; ^4^ Centre of Experimental Medicine and Surgery, Institute of Medical Sciences, Banaras Hindu University, Varanasi, India

**Keywords:** nitric oxide, visceral leishmaniasis, arginase, IL-10, TGF-beta

## Abstract

Nitric oxide (NO) is an anti-microbial effector of the innate immune system which plays major role in non-specific killing of various pathogens including protozoan parasites. However, due to subversion of the host’s immune processes by pathogens, suboptimal production of NO is frequently found in many infection models. Previous studies have shown suppressed NO production during *Leishmania donovani* infection, the causative agent of visceral leishmaniasis (VL). Availability of L-Arginine, a semi-essential amino acid is required for inducible nitric oxide synthase (iNOS) mediated NO production. However, arginase is another enzyme, which if expressed concomitantly, may strongly compete for L-Arginine, and suppress NO production by iNOS. In the present study, plasma nitrite and arginase levels were measured in VL patients before and after successful drug treatment, endemic and non-endemic healthy donors. We observed significantly lower NO levels in the plasma of VL patients as compared to endemic controls, which improved significantly post-treatment. Significantly elevated arginase activity was also observed in the plasma of VL patients, which may be associated with NO deficiency. VL patients also showed significantly higher levels of IL-10 and TGF-β, which are known to regulate expression of arginase in various immune cells. In vitro studies with human peripheral blood mononuclear cells (PBMCs) further corroborated the role of IL-10 and TGF-β in arginase mediated suppression of NO production.

## Introduction

Leishmaniasis represents a complex of diseases with a spectrum of severity ranging from cutaneous non-healing lesions to a potentially fatal visceralizing phenotype, depending upon the species of the *Leishmania* parasite involved and host-parasite interaction (Roberts, 2005). Visceral Leishmaniasis (VL, also known as Kala Azar), which the most severe and the visceralizing form is endemic in over 60 countries, out of which Bangladesh, Brazil, India, Nepal, and Sudan account for almost 90% of total incidences. In India, the state of Bihar reports majority of the cases, however, there has been a significant drop in the number of reported cases in last several years (Sundar and Agarwal, 2018; Kumar et al., 2020). The clinical outcome of these infections may depend upon several non-specific innate immune responses (Gurung and Kanneganti, 2015) and two functionally discrete immune responses, i.e., a pro-inflammatory T-helper (Th) type1 and an anti-inflammatory Th2 response (Miralles et al., 1994; Sundar et al., 1997). A robust Th1 response is a prerequisite for parasite elimination, while disease progression is invariably associated with differential activation and proliferation of the Th2 subset over Th1 as shown in murine infection models (Miralles et al., 1994). However, in human VL, a mixed Th1-Th2 phenotype is reported and despite presence of Th1 cytokines in the patients, a major role of the regulatory cytokines in VL etiology has been reported. Several studies have implicated IL-10 and TGF-β in VL pathogenesis (Sundar et al., 1997). In fact, past studies from our group and others have shown a direct role of IL-10 in the pathogenesis of human VL (Caldas et al., 2005; Gautam et al., 2011). Earlier thought to be a Th2 cytokine, IL-10 is produced by CD4+ FoxP3+ CD25+ regulatory T cells (Treg) as well as IFN- γ producing Treg cells (Tr1 cells) in humans (Moore et al., 2001; Spellberg and Edwards, 2001; Nylen et al., 2007; Chihara et al., 2016).

Nitric oxide (NO) is an innate immune effector which plays a crucial role in mediating killing of intracellular *Leishmania* parasites (Green et al., 1990). NO is produced by the activity of an enzyme, inducible nitric oxide synthase (iNOS/NOS2), the expression of which is commonly induced following pathogenic infections (Bogdan et al., 2000). Most commonly, activation of MAP kinases downstream of pattern recognition receptors like Toll-Like Receptors (TLRs) leads to a signaling cascade resulting in expression of the NOS2 protein (Jenner and Young, 2005; Pautz et al., 2010). Downstream of TLRs, transcription factor NFКβ is known to regulate induction of the NOS2 protein (Jenner and Young, 2005; Pautz et al., 2010). However, several other signaling cascades, if activated concomitantly, may cause significant repression of NOS2 expression even in the presence of its classical inducers and pathogens are known to exploit these mechanisms for their benefit. NO production is also known to be affected by arginase, an enzyme, which competes with NOS2 directly at the level of its substrate, L-arginine (Munder, 2009). NO production is strongly impaired in the presence of arginase, the expression of which is known to be induced by several immunosuppressive cytokines including IL-10 and TGF-β (Osorio et al., 2012). Based on previous studies about critical role of NO in controlling the expansion of the *Leishmania* parasites and providing tissue wide immunity in animal models (Olekhnovitch et al., 2014; Olekhnovitch and Bousso, 2015), we sought to investigate the status of NO response in human VL. We further examined the level of arginase activity and if it affected NO production in VL patients.

## Methods

### Study Subjects

Clinically confirmed cases of VL were recruited at the Kala-Azar Medical Research Center (KAMRC) in Muzaffarpur, Bihar State, India. The diagnosis was based on the clinical symptoms and detection of amastigotes in the splenic aspirates and/or positive serology for antibodies to the recombinant antigen, rK39 using a commercially available strip test (InBios Kala Azar Detect Rapid Test, USA). All patients included in the study were HIV negative, over 12 years of age and responded to Amphotericin B. Low hemoglobin, platelet count less than 40,000/μl and prothrombin time <5 s were additional criteria for exclusion. The endemic controls (EC) recruited into the study were serologically (rk39 dipstick) negative household contact of VL patients and were not asymptomatic. The patient and EC details have been summarized in (**Table 1**). Non-endemic healthy controls (NEHC) were from Varanasi and New Delhi and belonged to the age group 24–35 years.

**Table 1:** Demographic and clinical details of the study participants.

Variables	VL	EC
**N**	55	25
**Age (years)**	27.74 ± 17.00 (26)^a^	34.5 ± 12.5 (27)
**Sex (M/F)**	34/21	15/10
**Duration of illness (days)**	45.11 ± 38.38 (30)	N/A
**WBC (D-0)**	3718.87 ± 1569.18 (3400)	N/D
**WBC (D-Dis)**	10133.33 ± 4335.18 (9300)	N/D
**Hb (D-0)**	8.00 ± 1.78 (8)	N/A
**Hb (D-Dis)**	9.73 ± 1.61 (10)	N/A

N/A, not applicable; ND, not done; D-0, Day of admission; D-Dis, Day of discharge. ^a^Mean values ± SD of aggregated data are shown, and median values are in parentheses.

### Ethics Statement

The protocols used in this study were approved by the institutional human ethics review committees of the Institute of Medical Science, Banaras Hindu University, Varanasi. Written informed consents were obtained from the patients or their guardians.

### Human Peripheral Blood Sample Preparations

Five ml of venous blood was collected of heparinized tubes. Peripheral blood mononuclear cells (PBMCs) were isolated on a Ficoll-Hypaque density gradient as described earlier (Sharma et al., 2016). PBMCs were cultured in RPMI-1640 supplemented with L-glutamine, penicillin (100 U/ml), streptomycin (100 mg/ml), 10% heat-inactivated FBS and 1% human serum. Plasma was isolated by centrifuging 1 ml of blood at 2000 rpm for 10 min and was frozen at -80°C until subsequent use.

### Plasma Conditioning Experiments and Neutralization Assay

PBMCs were cultured at a density of 1X10^6^ cells/ml in 96 well flat-bottom culture plates (Nunc) at 37°C in 95% humidified air with 5% CO_2_. For plasma conditioning experiments, PBMCs were incubated in the RPMI-1640 medium containing 10% VL/EC or NEHC plasma for 12 h and subsequently treatments were performed. For neutralizing IL-10 and TGF-β in the VL plasma, monoclonal antibodies against human IL-10/TGF-β or corresponding isotype matched control immunoglobulin G (IgG) were added to a final concentration of 10 μg/ml. R&D systems, anti-IL-10 mouse IgG2b Clone # 23738 or anti- TGF-β mouse IgG_1_ clone # 1D11 and corresponding matched isotype controls, mouse IgG2B clone # 20116 or mouse IgG_1_ kappa clone # 11711 were used. All the experiments were performed in triplicates and the average value was taken.

### Enzyme Linked Immunosorbent Assay

Concentrations of the cytokines IL-10 or active TGF-β in the plasma or the cell-free culture supernatants were determined by commercially available ELISA kits as per the manufacturer’s instructions. (eBioscience, Cat. No. 88-7126; BioLegend, Cat. No. 437707).

### Measurement of Nitrite Production

The concentration of nitrite, the stable end-product of NO, in culture supernatants or plasma was determined by Griess’ reaction as described by Biswas et al. (2001) with slight modifications. Briefly, plasma was incubated with trichloroacetic acid before addition of the Griess’ reagent and centrifuged to collect the supernatant. The cell-free supernatant obtained from plasma or PBMC cultures were incubated with equal volume of Griess’ reagent for 30 min at 37°C. Readings were taken at 540 nm in a microplate reader (µQuant, BioTek Instruments Inc., VT, USA). Nitrite content was quantified by extrapolation from a sodium nitrite standard curve.

### Determination of Arginase Activity

Arginase activity in plasma and in the PBMC cultures was estimated as described earlier (Abebe et al., 2013). Briefly, PBMCs were lysed (0.1% Triton X-100, 25 mM Tris-HCl, 10 mM MnCl_2_) and subjected to heating at 56°C for 10 min to activate the enzyme. The processed lysate was incubated with 0.5 M L-arginine at 37°C for 30 min. The hydrolysis reaction was stopped with an acidified solution (H_2_SO_4_/H_3_PO_4_/H_2_O in a volumetric ratio 1:3:7). The concentration of urea, which is a product of L-arginine hydrolysis, was measured at 540 nm after addition of a-isonitrosopropiophenone (dissolved in 100% ethanol) and subsequent heating at 95°C for 30 min. For estimating arginase activity in the plasma, L-arginine hydrolysis was performed by directly incubating plasma with 0.5 M L-arginine and 10 mM MnCl_2_. The remaining steps were common to the protocol described for the PBMC lysates. The enzyme activity was defined in terms of units where one unit of enzyme corresponded to the amount of enzyme that catalyzes the generation of 1 μmol urea/min.

### Statistical Analyses

Data were analyzed using non-parametric two-tailed Mann-Whitney test. Wilcoxon signed rank test was applied to analyze paired samples. For correlation analysis the data were checked for Gaussian distribution using D’Agostino and Pearson omnibus normality test and subsequently subjected to Pearson rank test. Data are expressed as mean ± SD, and a p-value <0.05 was considered statistically significant. The analysis was performed using GraphPad Prism, version 5.01 (GraphPad, San Diego, CA).

## Results

### Human VL Patients Show Deficient NO Production During Active Phase of Disease

**Figure 1:**
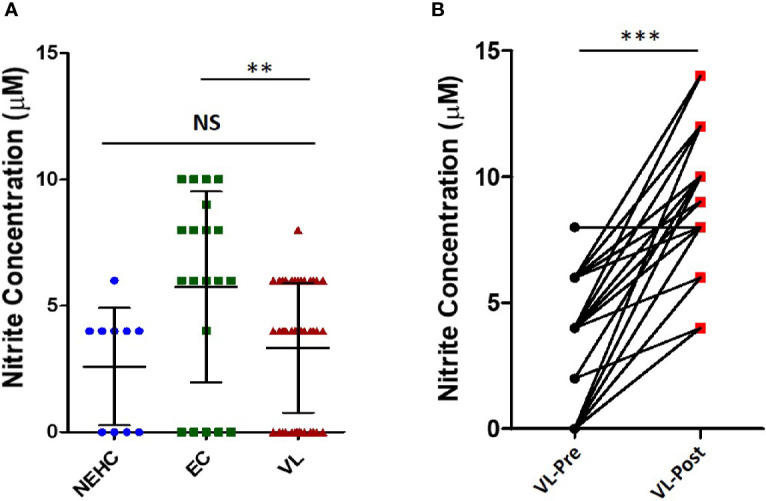
Nitrite levels in the VL plasma show improvement upon treatment. **(A)** Nitrite concentration in the plasma from VL patients (n=45) (circles), ECs (n=25) (squares), and NEHCs (n=10) (triangles) was determined by Griess’ assay. A two-tailed Mann-Whitney test was performed to compare the statistical differences between groups. **(B)** Same as in panel A, but a before and after plot for paired VL plasma samples (n=38), i.e., VL patients at presentation (circles) and after completion of treatment (squares). Wilcoxon signed rank test was applied for statistical significance. The data were earlier confirmed for Gaussian distribution (D’Agostino and Pearson omnibus normality test). **p < 0.01, ***p < 0.001; NS, not significant; VL, visceral leishmaniasis; EC, endemic control; NEHC, non-endemic healthy control.

Nitric oxide is a potent microbicidal molecule produced by the activity of the enzyme inducible nitric oxide synthase (iNOS) in response to infection (Bogdan et al., 2000). It is known to have strong anti-leishmanial activity. The levels of NO were measured in the plasma of VL patients at presentation (pre-treatment) and on the day of discharge (post-treatment) as well as in the plasma of healthy household contacts of the patients (ECs) and non-endemic healthy controls (NEHC). Plasma samples from a total of 45 VL patients, 20 ECs, and 10 NEHCs were estimated for NO. NEHCs as expected showed negligible levels of NO (2.6 ± 2.319 μM), however, no significant increase in the level of NO was observed in the plasma of VL patients, which ranged between 0 to 8μM (3.33 ± 2.558 μM). On the other hand, ECs showed slightly higher NO levels (5.75 ± 3.782 μM) as compared to VL patients (p=0.0031) (**Figure 1A**). Further, plasma samples collected from 38 VL subjects, pre- and post-treatment showed significant increase in the level of NO (2.947 ± 2.536 μM vs. 8.947 ± 2.567 μM; p<0.0001) (**Figure 1B**).

### VL Plasma Contains Factors Suppressing NO Production

**Figure 2:**
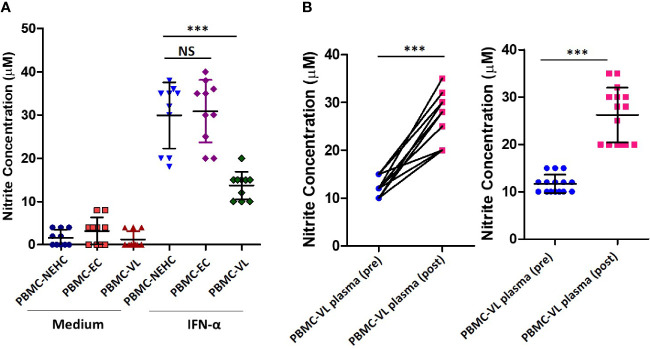
PBMCs derived from VL patients show reduced IFN-α-responsiveness for NO production. **(A)** PBMCs were derived from the VL patients (n=10), ECs (n=10) and non-endemic healthy controls (n=10) were incubated with or without IFN-α in complete RPMI medium for 48 h. Nitrite concentrations were measured in the cell-free supernatant. **(B)** PBMCs derived from NEHCs were incubated with before treatment (n=15) or after treatment VL plasma (n=15) for 12 h and subsequently activated with IFN-α (500 U/ml) for 48 h. Nitrite levels were measured in the cell-free supernatant. Data in panels A and B were analyzed using two-tailed Mann-Whitney test and Wilcoxon signed rank test respectively; **p<0.01, ***p<0.001; NS, not significant; PBMC, peripheral blood mononuclear cell; VL, visceral leishmaniasis; EC, endemic control; NEHC, non-endemic healthy control.

The above described results showed a generalized deficiency of nitrites in the plasma of VL patients (**Figure 1A**). We therefore hypothesized that PBMCs from the VL patients would be deficient in iNOS expression/activity. To test our hypothesis, PBMCs from NEHCs, ECs, and VL patients were incubated in medium alone or containing IFN-α (500 U/ml) and nitrite level was estimated as a measure of iNOS activity in cell-free supernatants after 48 h. IFN-α has been shown to induce NO production by human monocytes, and therefore was used as a stimulant for NO production (Sharara et al., 1997). PBMCs from NEHCs and ECs were found to produce comparable levels of nitrite (29.9 ± 7.622 vs. 30.9 ± 7.203 μM; p= 0.7302), however PBMCs from VL patients produced significantly lower nitrites as compared to either NEHCs (13.7 ± 3.199 vs. 29.9 ± 7.622 μM; p< 0.0003) or ECs (13.7 ± 3.199 vs. 30.9 ± 7.203 μM; p< 0.0002) (**Figure 2A**). To check the possibility that soluble factors in the VL plasma may be responsible for conditioning PBMCs towards low IFN-α responsivity in terms of NO production, PBMCs from NEHCs were isolated and conditioned with plasma from 15 VL patients, collected pre- and post-treatment. PBMCs from NEHCs were incubated with paired VL plasma for 12 h and then the PBMCs were stimulated with IFN-α for 48 h; cell-free supernatant was collected and assayed for nitrite level. Significantly higher level of NO was produced by PBMCs pre-incubated with plasma collected from VL patients post treatment, as compared to pre-treatment VL plasma (26.20 ± 5.797 vs. 11.67 ± 1.952 μM; p=0.0007) (**Figure 2B**).

### High Arginase Activity in the VL Plasma Accounts for Deficient NO Response

**Figure 3:**
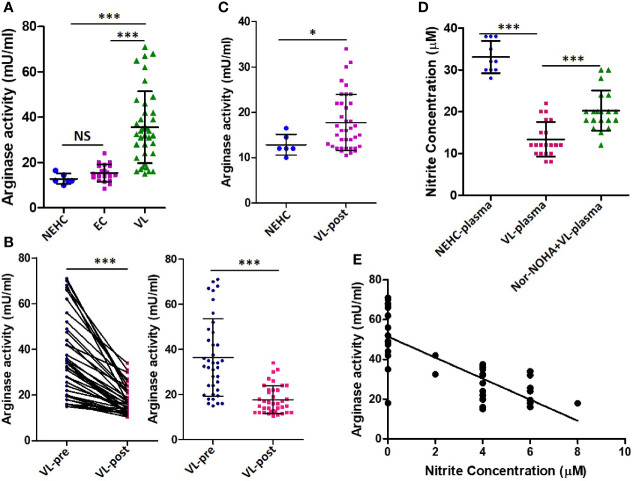
Arginase activity levels in the plasma of VL patients, ECs and NEHCs. **(A)** Whole blood was centrifuged to collect plasma from VL patients (VL; n=38) (circles), endemic controls (EC; n=20) (squares) or non-endemic healthy controls (NEHC; n=6) (triangles). Arginase activity in the plasma was measured by a colorimetric assay as detailed in the Methods section. A two-tailed Mann-Whitney test was performed to compare the statistical differences between groups. **(B)** Same as in panel A, but a before and after plot for paired VL plasma samples (n=38), i.e., VL patients at presentation (circles) and after completion of treatment (squares). Wilcoxon signed rank test was applied for statistical significance. **(C)** Comparison of arginase activity in the VL patients post-treatment with those of NEHCs. **(D)** PBMCs were isolated from the blood of NEHCs and incubated with VL plasma alone or in the presence of an arginase inhibitor, Nor-NOHA (n=20) for 12 h. Nitrite concentration was estimated in the cell-free supernatant after stimulation of the cells with IFN-a (500 U/ml) for 48 h as described in the Methods section. A two-tailed Mann-Whitney test was performed to compare the statistical differences between groups. **(E)** A correlation plot between arginase activity and plasma nitrite concentration. Mann-Whitney test; *p < 0.05, ***p < 0.001; NS, not significant; PBMC, peripheral blood mononuclear cell; VL, visceral leishmaniasis; EC, endemic control; NEHC, non-endemic healthy control.

Arginase is a competitive inhibitor of iNOS and interferes with NO generation when induced concomitantly (Rath et al., 2014). Moreover, arginase induction has been shown to mediate development of disease during *L. major* infection in a mouse model (Iniesta et al., 2005; Kropf et al., 2005). To investigate the possibility of arginase activity being a cause of deficient NO response in VL patients, arginase activity was measured in the patient plasma. Significantly higher arginase activity was found in the plasma of VL patients as compared to NEHC (35.60 ± 15.86 vs. 12.83 ± 2.295 mU/ml; p=0.0002) or EC plasma (35.60 ± 15.86 vs. 15.39± 3.775 mU/ml; p<0.0001) (**Figure 3A**). Upon pair-wise comparison (pre vs. post-treatment), statistically effective pairing for the samples was observed (Spearman rs=0.8031; p<0.0001) and plasma samples from treated patients showed highly significant reduction of arginase activity (36.46 ± 17.17 vs. 17.72 ± 6.201 mU/ml; p=0.0001) (**Figure 3B**). However, despite notable diminution of the arginase activity, plasma from treated patients still exhibited significantly higher arginase activity as compared to NEHCs (17.72 ± 6.201 vs. 12.83 ± 2.295 mU/ml; p=0.047) (**Figure 3C**). To further check if arginase inducing activity in the VL plasma accounted for the observed suppression of NO generation, PBMCs isolated from NEHCs were incubated for 12 h with VL plasma alone or in the presence of Nor-NOHA, an arginase inhibitor. Nitrite level was measured after 48 h of IFN-α treatment. Inhibition of the arginase activity in the VL plasma significantly restored the IFN-α -induced nitrite generation (13.40 ± 4.147 vs. 20.25 ± 1.07 μM; p=0.0001) (**Figure 3D**). However, arginase inhibition alone failed to restore the nitrite concentration to the control levels as the PBMCs incubated with arginase-inhibited VL plasma still produced significantly lesser nitrites upon IFN-α challenge as compared to control cells (20.25 ± 4.789 vs. 33.10 ± 3.843 μM; p<0.0001) (**Figure 3D**). With the observation of high levels of arginase activity in the VL plasma and deficiency of nitrite levels, we further explored if nitrite levels showed any empirical relationship with arginase activity. We plotted arginase activity in the VL plasma against corresponding nitrite values and performed a correlation analysis. A significant negative correlation was observed between arginase activity and nitrite levels (Pearson r=-0.7668; p<0.001) (**Figure 3E**).

### IL-10 and TGF-β in the VL Plasma Regulate NO Production *via* Arginase Induction

**Figure 4:**
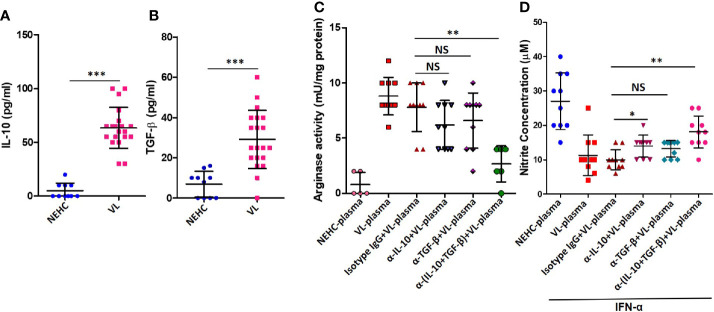
IL-10 and TGF-β in the VL plasma mediate arginase induction in PBMCs. **(A)** IL-10 cytokine levels were measured in the plasma of NEHCs (n=10) and VL patients (n=20) using an enzyme linked immunosorbent assay. **(B)** same as in A, but for active TGF-β. **(C)** PBMCs obtained from NEHCs were incubated with NEHC (n=5) or VL plasma, parental or neutralized either for IL-10 (anti-IL-10 mAb, 10 μg/ml) (n=10) or TGF-β (anti-TGF-β mAb, 10 μg/ml) (n=10) or both IL-10 and TGF-β (n=10) for 24 h and arginase activity in the whole cell lysates was estimated using a protocol detailed in the *Methods* section. **(D)** PBMCs conditioned with plasma, same as in **(C)**, were activated with IFN-α (500 U/ml) for 48 h and nitrite concentration in the cell-free supernatant was measured. Statistical significance was determined using Mann-Whitney test; *p<0.05, **p<0.01, ***p<0.001; NS, not significant; PBMC, peripheral blood mononuclear cell; VL, visceral leishmaniasis; NEHC, non-endemic healthy control.

IL-10 and TGF-β are known to mediated arginase induction in several models (Schreiber et al., 2009; Gordon and Martinez, 2010; Stempin et al., 2010; Makita et al., 2015). With an intention to account for the arginase inducing activity in the VL plasma, levels of IL-10 and active TGF-β were measured. Plasma from VL patients as compared to healthy controls showed significantly higher titers of IL-10 (63.5 ± 19.06 vs. 5 ± 7.071 pg/ml; p<0.0001) (**Figure 4A**) and TGF-β (29.10 ± 14.47 vs. 6.9 ± 6.402 pg/ml; p=0.0001) (**Figure 4B**). To ascertain if either of these cytokines mediated arginase induction, neutralization experiments were performed, where VL plasma was neutralized for IL-10 or TGF-β using functional-grade neutralizing antibodies. PBMCs isolated from NEHCs were incubated with VL plasma/VL plasma neutralized for IL-10 or TGF-β or both IL-10 and TGF-β for 48 h. Neutralization of either of these cytokines in the VL plasma resulted in significantly reduced arginase activity when compared with VL plasma alone (IL-10 neutralization: 8.8 ± 1.687 vs. 6.2 ± 2.201 mU/mg; p=0.012; TGF-β neutralization: 8.8 ± 1.687 vs. 6.6 ± 2.503 mU/mg; p=0.05), however the differences became statistically non-significant when values from isotype matched IgG control was used as reference (IL-10 neutralization: p=0.12; TGF-β neutralization: p=0.23) (**Figure 4C**). Further, when the VL plasma was neutralized for both IL-10 and TGF-β simultaneously, statistically significant reduction in the level of arginase activity was observed as compared to the isotype control (7.8 ± 2.20 vs. 2.67 ± 1.633 mU/mg; p=0.02) (**Figure 4C**), identifying both IL-10 and TGF-β as potential arginase inducing factors in the VL plasma. These results suggested that IL-10 and TGF-β present in the VL plasma mediated arginase induction. To further investigate if the reduced arginase activity upon IL-10/TGF-β neutralization also resulted in higher NO production, cells after plasma conditioning were further incubated with IFN-α for 48 h and nitrite level was measured in the cell-free supernatant. PBMCs conditioned with VL plasma produced significantly lesser nitrites as compared to those conditioned with NEHC plasma (11.3 ± 5.908 vs. 27 ± 8.233 μM; p=0.02) (**Figure 4D**). Conditioning with VL plasma neutralized for TGF-β alone did not show any significant increase in the nitrite levels as compared to PBMCs conditioned with VL plasma (12.0 ± 2.749 vs. 11.3 ± 5.908 μM; p=0.11). However, PBMCs conditioned with VL plasma neutralized for both IL-10 and TGF-β showed appreciable increment in the nitrite levels (17.6 ± 4.088 vs. 11.3 ± 5.908 μM; p=0.0011). Interestingly, PBMCs with VL plasma neutralized for IL-10 alone also produced marginally higher nitrite level which was borderline significant (13.3 ± 3.653 vs. 11.3 ± 5.908 μM; p=0.0474) (**Figure 4D**).

## Discussion

NO is an important effector molecule of the innate immune system and aids in the clearance of invading pathogens including protozoan parasites (James, 1995; Bogdan et al., 2000). Effective mounting of a regulated NO response can significantly limit the growth and spread of the pathogen. However, in the infected host, absence of a detectable NO response may favor rapid growth and spread of the pathogens (Olekhnovitch and Bousso, 2015). Animal models of *Leishmania* infection have demonstrated that NO is not only critical in restricting the growth of the parasite and providing tissue wide immunity but also in limiting the tissue damage due to infection (Olekhnovitch et al., 2014). With this background, we sought to look at the status of NO response in human VL. Nitrite estimation, which is classically used as a measure of NO expression, demonstrated very poor to almost non-existent NO expression in the VL patients during active phase of the disease. However, there was a marginal but statistically significant increase in the plasma nitrite levels post-treatment. On the other hand, ECs showed slightly higher plasma nitrite levels as compared to either NEHCs or VL patients. It must be noted that ECs were healthy household contacts of the VL patients recruited in the study and were serologically negative. Therefore, we could not attribute marginally higher nitrite levels in the plasma of ECs to an asymptomatic *L. donovani* infection. When the PBMCs isolated from VL patients were treated with IFN-α, they expressed significantly lower levels of NO as compared to the PBMCs either from ECs or NEHCs. We hypothesized that the PBMCs were probably conditioned or reprogrammed by certain soluble factors expressed at effectively higher titers in the VL patients as compared to ECs or NEHCs. To explore this possibility, plasma conditioning experiments were performed where PBMCs isolated from the NEHCs were incubated in the VL plasma and then treated with IFN-α. PBMCs incubated with plasma from ECs or NEHCs were used as controls. These experiments partially validated the hypothesis as PBMCs conditioned with VL plasma mounted significantly weaker NO response upon IFN-α treatment. It is noteworthy that VL patients produced extremely poor levels of NO despite having significantly higher levels of IFN-γ which is a potent inducer of NO. In a previous report, BALB/c derived peritoneal macrophages infected with *Leishmania donovani* showed impaired IFN-γ signaling due to deficient IFNγR1/R2 pairing (Sen et al., 2011). It is possible that such impairment of the IFN-γ signaling may contribute towards the absence of a robust NO response in human VL as well. Concomitant induction of arginase activity is one of the most common causes of a deficient NO response (Munder, 2009). Arginase is an enzyme which competes with NOS2 at the level of the common substrate, L-arginine. With this information in mind, we explored the possibility if the VL plasma either had high levels of arginase activity, factors with high arginase-inducing potential, or a combination of both. Plasma from VL patients showed significantly increased arginase activity during active phase of the disease which was brought down significantly upon treatment. However, plasma from VL subjects showed significantly higher arginase activity even after cure of the disease. It is important to mention that a small fraction of VL patients later develop post kala azar dermal leishmaniasis (PKDL), a sequel to VL (Singh et al., 2012). It may be worthwhile investigating if the residual arginase activity in the subjects upon cure of VL predisposed them to PKDL. Conditioning of the PBMCs isolated from NEHCs with VL plasma induced significantly higher levels of arginase activity. Interestingly, local increase of arginase activity has also been observed in the lesions of the patients of cutaneous leishmaniasis in Ethiopia (Abebe et al., 2012). Moreover, increased level of arginase activity has been shown to correlate with disease severity in HIV seropositive patients (Cloke et al., 2010). These findings were further corroborated using nor-NOHA, a commonly used arginase inhibitor. Cells conditioned with VL plasma produced significantly higher levels of NO when arginase activity was blocked with nor-NOHA. IL-10 and TGF-β are commonly associated with induction of arginase expression (Schreiber et al., 2009; Gordon and Martinez, 2010; Stempin et al., 2010; Makita et al., 2015), therefore we checked the level of these two cytokines in the VL plasma. In the hindsight, from our previous studies, we already knew that IL-10 is a strong susceptibility factor for human VL and neutralization of IL-10 through anti-IL10 antibody aids in resolution of the disease (Gautam et al., 2011). Both the cytokines were found to be expressed at significantly higher levels in the VL plasma. Neutralizing both IL-10 and TGF-β simultaneously led to highly significant reduction in the arginase inducing potential of the VL plasma, however, neutralization of IL-10 or TGF-β individually caused slight but statistically insignificant reduction, indicating role of both IL-10 and TGF-β in the regulation of arginase induction. Protein energy malnutrition is a major risk factor for VL (Malafaia, 2009), and is characterized by deficiency of essential amino acids. L-arginine is a semi-essential amino acid and apart from being a cellular building block, it is also required for NO production (Bogdan et al., 2000). In a mouse model of *Trypanosoma brucei* infection with a background of high arginase activity, L-arginine injection was shown to restore NO production and parasite killing (Gobert et al., 2000). Majority of the VL subjects included in the study were from financially poor households lacking means for proper nutrition. In the background of high arginase activity, relative deficiency of L-arginine in the diet may further predispose individuals to VL.

In summary, VL patients showed a generalized lack of NO production, which can be attributed to significantly increased arginase activity. IL-10 and TGF-βexpressed at significantly higher levels in the patients might suppress NO production by inducing arginase activity. Inhibition of arginase activity or neutralization of IL-10 and TGF-β caused significant restitution of the NO response.

## Data Availability Statement

The original contributions presented in the study are included in the article/supplementary material. Further inquiries can be directed to the corresponding author/s.

## Ethics Statement

The studies involving human participants were reviewed and approved by Institute of Medical Science, Banaras Hindu University, Varanasi. The patients/participants provided their written informed consent to participate in this study.

## Author Contributions

MK: Review of literature, Methodology, Data analysis and editing the draft. SSh: Sample recruitment, Methodology, Reviewing and editing of draft. RP: Conceptualization, Study design, Methodology, Data analysis and writing the manuscript. RK: Sample recruitment, Methodology, Reviewing and editing of draft, funding acquisition. SSu: Clinical resources, Clinical investigations and funding acquisition. SM: Conceptualization, Funding acquisition, Investigation, project administration and supervision, Validation, Editing and reviewing of draft. All authors contributed to the article and approved the submitted version.

## Funding

The work was supported by grants to SM: Department of Science and Technology, Science and Engineering Research Board (DST-SERB), grant no. SB/YS/LS-140/2014, Department of Science and Technology, Promotion of University Research and Scientific Excellence (DST-PURSE) SR/PURSE Phase 2/18 (G), the Government of India; SSu: NIH Tropical Medicine Research Centers (TMRC) grant no. U19 AI074321; RK: UGC seed grant and BHU-IoE grant.

## Conflict of Interest

RK was employed by Thermo Fisher Scientific.

The remaining authors declare that the research was conducted in the absence of any commercial or financial relationships that could be construed as a potential conflict of interest.

## References

[B1] Abebe T. Hailu A. Woldeyes M. Mekonen W. Bilcha K. Cloke T.. (2012). Local increase of arginase activity in lesions of patients with cutaneous leishmaniasis in Ethiopia. PloS Negl. Trop. Dis. 6, e1684. 10.1371/journal.pntd.0001684 22720104 PMC3373636

[B2] Abebe T. Takele Y. Weldegebreal T. Cloke T. Closs E. Corset C.. (2013). Arginase activity - a marker of disease status in patients with visceral leishmaniasis in ethiopia. PloS Negl. Trop. Dis. 7, e2134. 10.1371/journal.pntd.0002134 23556019 PMC3610885

[B3] Biswas S. K. Sodhi A. Paul S. (2001). Regulation of nitric oxide production by murine peritoneal macrophages treated in vitro with chemokine monocyte chemoattractant protein 1. Nitric. Oxide 5, 566–579. 10.1006/niox.2001.0370 11730364

[B4] Bogdan C. Rollinghoff M. Diefenbach A. (2000). The role of nitric oxide in innate immunity. Immunol. Rev. 173, 17–26. 10.1034/j.1600-065x.2000.917307.x 10719664

[B5] Caldas A. Favali C. Aquino D. Vinhas V. van W. J. Brodskyn C.. (2005). Balance of IL-10 and interferon-gamma plasma levels in human visceral leishmaniasis: implications in the pathogenesis. BMC Infect. Dis. 5, 113. 10.1186/1471-2334-5-113 16364177 PMC1343567

[B6] Chihara N. Madi A. Karwacz K. Awasthi A. Kuchroo V. K. (2016). Differentiation and Characterization of Tr1 Cells. Curr. Protoc. Immunol. 1 (113), 3.27.1–3.27.10. 10.1002/0471142735.im0327s113 PMC5933847 27038462

[B7] Cloke T. E. Garvey L. Choi B. S. Abebe T. Hailu A. Hancock M.. (2010). Increased level of arginase activity correlates with disease severity in HIV-seropositive patients. J. Infect. Dis. 202, 374–385. 10.1086/653736 20575659 PMC4663662

[B8] Gautam S. Kumar R. Maurya R. Nylen S. Ansari N. Rai M.. (2011). IL-10 neutralization promotes parasite clearance in splenic aspirate cells from patients with visceral leishmaniasis. J. Infect. Dis. 204, 1134–1137. 10.1093/infdis/jir461 21881130 PMC3164427

[B9] Gobert A. P. Daulouede S. Lepoivre M. Boucher J. L. Bouteille B. Buguet A.. (2000). L-Arginine availability modulates local nitric oxide production and parasite killing in experimental trypanosomiasis. Infect. Immun. 68 (8), 4653–4657. 10.1128/iai.68.8.4653-4657.2000 10899869 PMC98402

[B10] Gordon S. Martinez F. O. (2010). Alternative activation of macrophages: mechanism and functions. Immunity 32, 593–604. 10.1016/j.immuni.2010.05.007 20510870

[B11] Green S. J. Mellouk S. Hoffman S. L. Meltzer M. S. Nacy C. A. (1990). Cellular mechanisms of nonspecific immunity to intracellular infection: cytokine-induced synthesis of toxic nitrogen oxides from L-arginine by macrophages and hepatocytes. Immunol. Lett. 25 (1-3), 15–19. 10.1016/0165-2478(90)90083-3 2126524

[B12] Gurung P. Kanneganti T. D. (2015). Innate immunity against Leishmania infections. Cell. Microbiol. 17, 1286–1294. 10.1111/cmi.12484 26249747 PMC4698274

[B13] Iniesta V. Carcelén J. Molano I. Peixoto P. M. Redondo E. Parra P.. (2005). Arginase I induction during Leishmania major infection mediates the development of disease. Infect. Immun. 73 (9), 6085–6090. 10.1128/IAI.73.9.6085-6090.2005 16113329 PMC1231060

[B14] James S. L. (1995). Role of nitric oxide in parasitic infections. Microbiol. Rev. 59 (4), 533–547. 10.1128/MR.59.4.533-547.1995 8531884 PMC239385

[B15] Jenner R. G. Young R. A. (2005). Insights into host responses against pathogens from transcriptional profiling. Nat. Rev. Microbiol. 3, 281–294. 10.1038/nrmicro1126 15806094

[B16] Kropf P. Fuentes J. M. Fähnrich E. Arpa L. Herath S. Weber V.. (2005). Arginase and polyamine synthesis are key factors in the regulation of experimental leishmaniasis in vivo. FASEB J. 19 (8), 1000–1002. 10.1096/fj.04-3416fje 15811879

[B17] Kumar V. Mandal R. Das S. Kesari S. Dinesh D. S. Pandey K.. (2020). Kala-azar elimination in a highly-endemic district of Bihar, India: A success story. PloS Negl. Trop. Dis. 14 (5), e0008254. 10.1371/journal.pntd.0008254 32365060 PMC7224556

[B18] Makita N. Hizukuri Y. Yamashiro K. Murakawa M. Hayashi Y. (2015). IL-10 enhances the phenotype of M2 macrophages induced by IL-4 and confers the ability to increase eosinophil migration. Int. Immunol. 27 (3), 131–141. 10.1093/intimm/dxu090 25267883

[B19] Malafaia G. (2009). Protein-energy malnutrition as a risk factor for visceral leishmaniasis: a review. Parasite Immunol. 31, 587–596. 10.1111/j.1365-3024.2009.01117.x 19751470

[B20] Miralles G. D. Stoeckle M. Y. McDermott D. F. Finkelman F. D. Murray H. W. (1994). Th1 and Th2 cell-associated cytokines in experimental visceral leishmaniasis. Infect. Immun. 62, 1058–1063. 10.1128/IAI.62.3.1058-1063 8112840 PMC186224

[B21] Moore K. W. de Waal Malefyt R. Coffman R. L. O’Garra A. (2001). Interleukin-10 and the interleukin-10 receptor. Ann. Rev. Immunol. 19, 683–765. 10.1146/annurev.immunol.19.1.683 11244051

[B22] Munder M. (2009). Arginase: an emerging key player in the mammalian immune system. Br. J. Pharmacol. 158, 638–651. 10.1111/j.1476-5381.2009.00291.x 19764983 PMC2765586

[B23] Nylen S. Maurya R. Eidsmo L. Manandhar K. D. Sundar S. Sacks D. (2007). Splenic accumulation of IL-10 mRNA in T cells distinct from CD4+CD25+ (Foxp3) regulatory T cells in human visceral leishmaniasis. J. Exp. Med. 204 (4), 805–817. 10.1084/jem.20061141 17389235 PMC2118563

[B24] Olekhnovitch R. Bousso P. (2015). Induction, Propagation, and Activity of Host Nitric Oxide: Lessons from Leishmania Infection. Trends Parasitol. 31, 653–664. 10.1016/j.pt.2015.08.001 26440786

[B25] Olekhnovitch R. Ryffel B. Muller A. J. Bousso P. (2014). Collective nitric oxide production provides tissue-wide immunity during Leishmania infection. J. Clin. Invest 124, 1711–1722. 10.1172/JCI72058 24614106 PMC3973105

[B26] Osorio E. Y. Zhao W. Espitia C. Saldarriaga O. Hawel L. Byus C. V.. (2012). Progressive visceral leishmaniasis is driven by dominant parasite-induced STAT6 activation and STAT6-dependent host arginase 1 expression. PloS Pathog. 8, e1002417. 10.1371/journal.ppat.1002417 22275864 PMC3261917

[B27] Pautz A. Art J. Hahn S. Nowag S. Voss C. Kleinert H. (2010). Regulation of the expression of inducible nitric oxide synthase. Nitric. Oxide 23, 75–93. 10.1016/j.niox.2010.04.007 20438856

[B28] Rath M. Muller I. Kropf P. Closs E. I. Munder M. (2014). Metabolism via Arginase or Nitric Oxide Synthase: Two Competing Arginine Pathways in Macrophages. Front. Immunol. 5:3389/fimmu.2014.00532:532. 10.3389/fimmu.2014.00532 PMC4209874 25386178

[B29] Roberts M. T. (2005). Current understandings on the immunology of leishmaniasis and recent developments in prevention and treatment. Br. Med. Bull. 75-76, 115–130. 10.1093/bmb/ldl003 16847165

[B30] Schreiber T. Ehlers S. Heitmann L. Rausch A. Mages J. Murray P. J.. (2009). Autocrine IL-10 induces hallmarks of alternative activation in macrophages and suppresses antituberculosis effector mechanisms without compromising T cell immunity. J. Immunol. 183 (2), 1301–1312. 10.4049/jimmunol.0803567 19561100 PMC2735238

[B31] Sen S. Roy K. Mukherjee S. Mukhopadhyay R. Roy S. (2011). Restoration of IFNγR subunit assembly, IFNγ signaling and parasite clearance in Leishmania donovani infected macrophages: role of membrane cholesterol. PloS Pathog. 7 (9), e1002229. 10.1371/journal.ppat.1002229 21931549 PMC3169561

[B32] Sharara A. I. Perkins D. J. Misukonis M. A. Chan S. U. Dominitz J. A. Weinberg J. B. (1997). Interferon (IFN)-alpha activation of human blood mononuclear cells in vitro and in vivo for nitric oxide synthase (NOS) type 2 mRNA and protein expression: possible relationship of induced NOS2 to the anti-hepatitis C effects of IFN-alpha in vivo. J. Exp. Med. 186, 1495–1502. 10.1084/jem.186.9.1495 9348307 PMC2199112

[B33] Sharma S. Davis R. E. Srivastva S. Nylen S. Sundar S. Wilson M. E. (2016). A Subset of Neutrophils Expressing Markers of Antigen-Presenting Cells in Human Visceral Leishmaniasis. J. Infect. Dis. 214, 1531–1538. 10.1093/infdis/jiw394 27601622 PMC5091370

[B34] Singh R. P. Picado A. Alam S. Hasker E. Singh S. P. Ostyn B.. (2012). Post-kala-azar dermal leishmaniasis (PKDL) in visceral leishmaniasis-endemic communities in Bihar, India. Trop. Med. Int. Health. 10.1111/tmi.12044 23279744

[B35] Spellberg B. Edwards J. E. Jr. (2001). Type 1/Type 2 immunity in infectious diseases. Clin. Infect. Dis. 32, 76–102. 10.1086/317537 11118387

[B36] Stempin C. C. Dulgerian L. R. Garrido V. V. Cerban F. M. (2010). Arginase in parasitic infections: macrophage activation, immunosuppression, and intracellular signals. J. Biomed. Biotechnol. 2010:683485. 10.1155/2010/683485 20029630 PMC2792949

[B37] Sundar S. Agarwal D. (2018). Visceral Leishmaniasis – Optimum Treatment Options in Children. Pediatr. Infect. Dis. J. 37 (5), 492–494. 10.1097/INF.0000000000001885 29280784 PMC5990428

[B38] Sundar S. Reed S. G. Sharma S. Mehrotra A. Murray H. W. (1997). Circulating T helper 1 (Th1) cell- and Th2 cell-associated cytokines in Indian patients with visceral leishmaniasis. Am. J. Trop. Med. Hyg. 56, 522–525. 10.4269/ajtmh.1997.56.522 9180602

